# Does Abnormal Preoperative Coagulation Status Lead to More Perioperative Blood Loss in Spinal Deformity Correction Surgery?

**DOI:** 10.3389/fsurg.2022.841680

**Published:** 2022-07-22

**Authors:** Zheng Li, Bin Yu, Jianguo Zhang, Jianxiong Shen, Yipeng Wang, Guixing Qiu, Xinqi Cheng

**Affiliations:** Department of Orthopaedic Surgery, Peking Union Medical College Hospital, Chinese Academy of Medical Sciences and Peking Union Medical College, Beijing, China

**Keywords:** spinal deformity, blood loss, preoperative coagulation status, activated partial thromboplastin time, estimated blood loss

## Abstract

This study aims to analyze the potential association between the preoperative coagulation status and perioperative blood loss in spinal deformity correction surgery. The preoperative coagulation status and estimated blood loss (EBL) during operation, postoperative wound drainage, and allogeneic transfusion during and after operation were recorded and analyzed. Among the 164 patients, 26 had a longer prothrombin time (PT), 13 had a lower fibrinogen level, 55 had a longer activated partial thromboplastin time (APTT), and 2 had a longer thrombin time (TT), and the platelet count (PLT) was all normal or higher than the normal level. The mean EBL per surgical level was 77.8 ml (range, 22–267 ml), and the mean drainage per surgical level was 52.7 ml (range, 7–168 ml). Fifty-five patients and 12 patients underwent allogeneic transfusion during and after the operation, respectively. The differences in EBL per surgical level, mean drainage per surgical level, the occurrences of allogeneic transfusion during and after operation between the patients with a longer PT, lower fibrinogen level, longer APTT or longer TT, and the normal controls were not significant (all *P*’s > 0.05). The Spearman correlation analysis showed that there was no correlation between PT, fibrinogen, APTT, TT or PLT with EBL per surgical level, mean drainage per surgical level, or allogeneic transfusion during and after the operation (all *P*’s > 0.05). The abnormal preoperative coagulation status but not hemophilia does not lead to more perioperative blood loss or a higher rate of perioperative allogeneic transfusion in spinal deformity correction surgery.

## Introduction

Scoliosis correction surgery is major surgery with a large estimated blood loss (EBL) during the operation (1–6). Normal clotting requires normal coagulation status of the coagulation factors, and patients with obvious abnormal coagulopathy may experience more perioperative blood loss and a high incidence of bleeding complications in scoliosis correction surgery (7–10). In 2005, Ho et al. found a high prevalence of coagulation abnormalities among their adolescent idiopathic scoliosis (AIS) patients (9). In Ryan et al.’s study, children with scoliosis also have a higher prevalence of preoperative coagulation abnormalities compared with healthy patients (10), so the coagulation status test should be carried out prior to the surgery. Carling et al. reported that total bleeding volume in AIS correction surgery correlated significantly with preoperative fibrinogen concentration (11). Geck et al. also reported that preoperative fibrinogen levels exhibited a significant negative logarithmic correlation with total blood loss in their AIS correction surgeries (12). However, there are few studies focusing on the association between abnormal coagulation and blood loss in scoliosis correction surgery. In our department, preoperative coagulation screening tests are routinely performed in patients undergoing spinal deformity correction surgery. Our study analyzed whether there were potential associations between preoperative coagulation status and perioperative blood loss.

## Materials and Methods

After obtaining approval from the institutional review board, we performed the current study. One hundred and sixty-four patients, 61 males and 103 females with an average age of 14.6 years (range, 10–20), who underwent scoliosis correction surgery in our hospital from May 2010 to April 2012 were included. The medical records, radiographic parameters of the deformity, and laboratory blood tests were recorded, and laboratory blood tests were performed 2 days before the operation. The preoperative coagulation status [platelet count (PLT), prothrombin time (PT), fibrinogen, activated partial thromboplastin time (APTT), thrombin time (TT)] and EBL during operation, postoperative wound drainage, and allogeneic transfusion during and after operation were also recorded and analyzed. None of the patients had received a prophylactic regimen or were treated with aspirin.

The normal limits of the PLT, PT, fibrinogen, APTT, and TT were 10–35 G/L,10.4–12.6 s, 1.80–3.50 g/L, 22.7–31.8 s and 14.0–21.0 s, respectively. We defined the abnormal coagulative from these numerical ranges.

### Statistics

SPSS 17.0 for Windows was used in this study; a *t*-test or *U*-test was used for comparison; and the correlation between PT, international normalized ratio (INR), fibrinogen, APTT, TT or PLT, EBL per surgical level, mean drainage per surgical level, or allogeneic transfusion during and after the operation was analyzed by Spearman correlation analysis.

## Results

The etiology of the deformity included idiopathic scoliosis in 63 patients, congenital scoliosis in 70 patients, neuromuscular scoliosis in 20 patients, neurofibromatosis scoliosis in five patients, and Marfan syndrome scoliosis in six patients. The mean Cobb angle of the major curve, fusion level, operation time, total EBL, EBL per surgical level, total wound drainage, and wound drainage per surgical level are listed in Table 1. Fifty-five patients and 12 patients underwent allogeneic transfusion during and after the operation, respectively. The details of allogeneic transfusion are listed in Table 1.

**Table 1:** Details of the Cobb angle of the major curve, fusion level, operation time, EBL, and allogeneic transfusion.

	Min	Max	Mean	SD
Cobb angle of major curve (degree)	30.0	158.0	64.2	25.2
Operation time (h)	1.3	8.5	4.1	1.3
Fusion level (vertebra)	5.0	17.0	10.9	3.0
Total EBL	200.0	2,500.0	809.6	415.4
EBL per level	22.2	266.7	77.8	43.7
Total drainage (ml)	100.0	1,850.0	557.0	268.6
Drainage per level (ml)	7.0	168.2	52.7	24.9
RBC transfusion (during operation) (U)	0	8.0	0.96	1.59
Plasma transfusion (during operation) (ml)	0	800.0	104. 3	190.4
RBC transfusion (after operation) (U)	0	9.0	0.4	1.3
Plasma transfusion (after operation) (ml)	0	800.0	25.5	106.4

*EBL, estimated blood loss; RBC, red blood cells.*

The results of the blood screening test for the coagulation factors are listed in Table 2.

**Table 2:** Details of the blood screening test of the coagulation factors.

	Min	Max	Mean	SD
PT (10.4–12.6 s)	10.70	15.30	12.07	0.702
INR (0.86–1.14)	0.89	1.30	1.03	0.06
Fibrinogen (1.80–3.50 g/L)	1.16	3.84	2.34	0.45
APTT (22.7–31.8 s)	22.60	42.40	30.50	4.22
TT (14.0–21.0 s)	15.70	22.80	18.17	1.08
PLT (100–350 G/L)	124.00	475.00	256.20	54.18

*PT, prothrombin time; INR, international normalized ratio; APTT, activated partial thromboplastin time; TT, thrombin time; PLT, platelet count.*

Among the 164 patients, 26 had a longer PT, 13 had a lower fibrinogen level, 55 had a longer APTT (30 with 3 s or longer than normal, 14 with 5 s or longer than normal), and two had a longer TT, and the PLT values were all higher than the normal level. Among the 26 patients with a longer PT, six also had a longer INR. Each of the 17 patients had both longer PT and APTT and a lower fibrinogen level. Five patients had a longer APTT and a lower fibrinogen level.

The differences in EBL per surgical level, mean drainage per surgical level, the occurrences of allogeneic transfusion during and after the operation between the patients with a longer PT time, lower fibrinogen level, longer APTT or longer TT, and the normal controls were not significant (all *P*’s > 0.05). The patients with both abnormalities of the PT and APTT did not have more EBL per surgical level, more mean drainage per surgical level, and more occurrences of allogeneic transfusion during and after the operation compared to the other patients.

The Spearman correlation analysis showed that there was no correlation between PT, INR, fibrinogen, APTT, TT or PLT with EBL per surgical level, mean drainage per surgical level, or allogeneic transfusion during and after the operation (all *P*’s > 0.05) (Table 3).

**Table 3:** Spearman correlation analysis.

Spearman	EBL per surgical level	Mean drainage per surgical level	Allogeneic transfusion during operation	Allogeneic transfusion after operation
PT	0.564	0.831	0.621	0.120
INR	0.877	0.284	0.587	0.919
Fibrinogen	0.091	0.907	0.127	0.841
APTT	0.780	0.364	0.919	0.809
TT	0.162	0.051	0.304	0.606
PLT	0.453	0.186	0.528	0.269

*PT, prothrombin time; INR, international normalized ratio; APTT, activated partial thromboplastin time; TT, thrombin time; PLT, platelet count.*

## Discussion

The most common, routinely collected coagulation markers are APTT, PT, and PLT (10, 13, 14). This has important implications for clinical practice because the identification of hemostatic defects prior to surgery may allow affected patients to be targeted with specific interventions to reduce the risk of bleeding (11, 15, 16).

In the literature, some doctors have reported a higher rate of coagulation abnormalities in scoliosis patients (9, 10, 17). In Ho et al.’s report, eight patients (25%) had a prolonged APTT, while the INR and fibrinogen were all normal (9). Ryan et al. compared 165 pediatric scoliosis patients with 175 controls and found that scoliosis patients had a significantly greater prevalence of abnormal coagulation screening tests (67%) compared with controls (43%; OR, 2.6; 95% CI, 1.5–4.6, *P* < 0.001). In our series, 74 patients had abnormal results from the screening coagulation tests, and 65 patients had prolonged PT, APTT, or lower fibrinogen, which was similar to Ho’s report. In our series, 26 (15.8%) patients had prolonged PT, of which six patients also had prolonged INR, and 55 (33.5%) patients had prolonged APTT. In Ryan et al.’s study, 62.6% (103/165) of scoliosis cases had an abnormal PT, and they thought that their results might be heavily influenced by the PT results. However, in the current study, APTT abnormalities were more often than others.

Two pathways lead to the formation of a fibrin clot: the intrinsic and extrinsic pathways. The common point in both pathways is the activation of factor X to factor Xa. Factor Xa activates prothrombin (factor II) to thrombin (factor IIa). The PT is an assay designed to measure activities of the extrinsic pathway of coagulation, while the APTT is used to assess for defects in the intrinsic pathway of coagulation. The most common measure of PT is to divide the time of coagulation of a patient’s blood by that of a known standard, and this value is referred to as the INR (10, 14, 18).

In Carling et al.’s study, there was no correlation between the mean total bleeding volume and PLT, APTT, or PT (*P* = 0.61, 0.46, and 0.57, respectively). Also, the differences in PLT, APTT, or PT between patients with an extensive transfusion or bleeders with the controls were none significant (all *P*’s > 0.05), too. In Ryan et al.’s report, the mean values of PT, APTT, and TT were all significantly higher in the scoliosis cases compared with those in non-scoliosis controls (*P* < 0.001, after adjusting for age and gender). However, they did not analyze the association between these abnormalities and blood loss during the operations (10). Ialenti et al. studied the effects of PT/PTT on intraoperative blood loss (IOBL) and found no significant associations between PT/PTT and IOBL (5). In 2015, Li et al. performed a study to analyze the preoperative factors and IOBL in female AIS patients. Of the 161 patients, the PLT, PT, and TT were not found to be significantly related to IOBL, while the APTT was found to be significantly associated with IOBL (19). Horlocker et al. found that the screening tests of PT and APTT gave limited information about how much a patient will bleed during and after the procedure (7). Shaw et al. analyzed 48 consecutive referrals for abnormal PTs, partial thromboplastin times, or closure times obtained as preprocedural screens and found only 9 patients (19%) had a possible or true mild bleeding disorder. Thus, they concluded that the usefulness of using PT and APTT in diagnosing bleeding disorders in pediatric patients preoperatively was limited (20). In the current series, the differences in blood loss and allogeneic transfusion between the patients with a longer PT time, lower fibrinogen level, longer APTT or longer TT, and the normal controls were none significant (all *P*’s > 0.05) neither did the patients with both abnormalities of the PT and APTT. Also, the correlation analysis did not show a significant association between PT, APTT, perioperative blood loss, and allogeneic transfusion. The current results were similar to Carling, Ialenti, Horlocker, and Shaw et al.’s studies, while there was a little difference from Li et al.’s study.

Fibrinogen is a key protein in the coagulation cascade, which is one of the first coagulation factors consumed during bleeding. During coagulation, thrombin lyses the soluble fibrinogen into the insoluble fibrin net as the final step of clotting (7, 16, 21).

Carling et al. studied the preoperative fibrinogen plasma concentration to investigate the potential association between fibrinogen, bleeding, and transfusion requirements after scoliosis surgery. Among their 82 patients, the mean total bleeding volume was correlated significantly with preoperative fibrinogen concentration (*r* = 0.31, *P* = 0.005). According to their study, a lower preoperative fibrinogen plasma concentration was associated with bleeding >1920 ml and extensive transfusion (both *P*’s = 0.002). They also found that an individual patient with a fibrinogen value above 2.8 g/L is unlikely to bleed extensively, while lower fibrinogen concentration patients have an increased risk of high bleeding volume or transfusion rate. They concluded that the preoperative measurement of the fibrinogen concentration provides more information about bleeding volume and transfusion requirements than standard screening tests (11). Geck et al. also performed a study focused on the association between preoperative fibrinogen, bleeding, and transfusion requirements. In their 110 AIS patients, the preoperative fibrinogen was significantly correlated with the total bleeding volume and transfusion (12). In Li et al.’s report, fibrinogen was found to be significantly associated with IOBL (20). The current study did not find significant differences in blood loss or allogeneic transfusion between patients with lower and normal preoperative fibrinogen, and there was no significant association between preoperative fibrinogen, perioperative blood loss, and allogeneic transfusion. These results were different from the above-mentioned studies.

However, there were also some limitations of the current study: first, although the sample was relatively large, it was still small; second, it was a single-center case series, and intrinsic selective bias was inevitable; third, the results would have been more convincing if there were normal controls to match the scoliosis patients; last, some doctors also used thromboelastography to assess the coagulation status of the scoliosis patients (8, 12), while in the current study, this was not analyzed.

In summary, from the limited number of scoliosis patients, the present study found that patients with abnormal coagulation screening tests did not have more blood loss during and after the operation and a high possibility of perioperative allogeneic transfusion. Associations between these abnormalities and perioperative blood loss and allogeneic transfusion were not found, either. Therefore, abnormal preoperative coagulation status but not hemophilia does not appear to lead to more perioperative blood loss or a higher rate of perioperative allogeneic transfusion in spinal deformity correction surgery. In the future, further studies are needed to assess the clinical significance of our results.

## Data Availability

The original contributions presented in the study are included in the article/Supplementary Material, further inquiries can be directed to the corresponding author/s.
